# ^13^C and ^15^N assimilation and organic matter translocation by the endolithic community in the massive coral *Porites lutea*

**DOI:** 10.1098/rsos.171201

**Published:** 2017-12-06

**Authors:** Laddawan Sangsawang, Beatriz Estela Casareto, Hideo Ohba, Hung Manh Vu, Aussanee Meekaew, Toshiyuki Suzuki, Thamasak Yeemin, Yoshimi Suzuki

**Affiliations:** 1 Department of Environment and Energy Systems, Graduate Schools of Science and Technology, Shizuoka University, Shizuoka, Japan; 2 Marine and Coastal Resources Research and Development Center, the Eastern Gulf of Thailand, Rayong Province, Thailand; 3 Research Institute of Green Science and Technology, Shizuoka University, Shizuoka, Japan; 4 Institute of Marine Environment and Resources, Vietnam Academy of Science and Technology (VAST), Hanoi, Vietnam; 5 Marine Biodiversity Research Group, Faculty of Science, Ramkhamhaeng University, Bangkok, Thailand

**Keywords:** endolithic algae, primary production, nitrogen fixation, translocation, *Ostreobium quekettii*, *Porites lutea*

## Abstract

Corals evolved by establishing symbiotic relationships with various microorganisms (the zooxanthellae, filamentous algae, cyanobacteria, bacteria, archaea, fungi and viruses), forming the ‘coral holobiont'. Among them, the endolithic community is the least studied. Its main function was considered to be translocation of photo-assimilates to the coral host, particularly during bleaching. Here, we hypothesize that (i) endolithic algae may show similar primary production rates in healthy or bleached corals by changing their pigment ratios, and therefore that similar production and translocation of organic matter may occur at both conditions and (ii) diazotrophs are components of the endolithic community; therefore, N_2_ fixation and translocation of organic nitrogen may occur. We tested these hypotheses in incubation of *Porites lutea* with ^13^C and ^15^N tracers to measure primary production and N_2_ fixation in coral tissues and endoliths. Assimilation of the ^13^C atom (%) was observed in healthy and bleached corals when the tracer was injected in the endolithic band, showing translocation in both conditions. N_2_ fixation was found in coral tissues and endolithic communities with translocation of organic nitrogen. Thus, the endolithic community plays an important role in supporting the C and N metabolism of the holobiont, which may be crucial under changing environmental conditions.

## 1. Introduction

Stony corals or reef-building corals are the most important components of the coral reef ecosystems that are distributed in tropical areas of the world's oceans. Corals evolved to adapt to oligotrophic environments by establishing symbiotic relationships with a variety of microbes forming the so-called ‘coral holobiont' [1–4]. In addition to the well-known symbiotic algae zooxanthellae, other associated organisms play crucial roles in environmental adaptation of the coral holobiont by providing organic and inorganic carbon [5] and organic nitrogen [6,7], and protecting the coral host from pathogens [8–10].

Recent changes in environmental conditions, such as increased sea surface temperature, high irradiance and low water quality, are promoting coral bleaching and disease. However, the coral holobiont has the capacity to acclimatize (to some extent) to these environmental changes by rapidly altering the population of associated microbes and their functioning in a dynamic way. The ‘coral probiotic hypothesis' was proposed to explain this concept [11].

Among associated microbes, the endolithic community is one of the less studied entities within the coral holobiont. Members of this community appear in remarkably high numbers in massive and encrusting corals [12,13]. Previous studies had shown that the endolithic community consists of filamentous algae, especially chlorophytes of the genus *Ostreobium*, cyanobacteria and fungi [14–16]. More recently, molecular studies revealed the presence of several bacteria, such as Firmicutes, Actinobacteria, Proteobacteria and Chlorobi, including nitrogen fixers and green sulfur bacteria [17]. Several functions were attributed to this community, such as providing potential nutrient sources [18] and photoprotection for the coral host [19,20], particularly during bleaching events. Fine & Loya [18] showed increasing ^14^C activity with time in coral tissues after addition of ^14^C inside the endolithic band of bleached corals; however, they did not evaluate this process in terms of the organic matter synthesized and transferred.

In this study, we wished to determine how much the endolithic community fixes organic carbon and whether the endolithic community is capable of fixing atmospheric nitrogen and translocating it to the coral host. We also wanted to know what percentage of the synthesized organic C is translocated to the coral tissue under the healthy and stressful condition, and what the responses are of photosynthetic pigments of the endolithic community under higher illumination when the coral is bleached.

We hypothesized that (i) endolithic algae keep their photosynthetic performance at similar levels in healthy or bleached corals; therefore, translocation may occur similarly under both conditions; (ii) cyanobacteria and other N_2_ fixers are components of the endolithic community; therefore, N_2_ fixation may occur in this layer and organic nitrogen-rich compounds may be transferred together with organic carbon; and (iii) the endolithic community may acclimatize to changes of illumination under bleached coral conditions by rapid changes of their pigments and pigment ratios.

In this study, we provide a quantification of the primary production and nitrogen fixation of the endolithic community and the coral tissues in terms of organic carbon and nitrogen; we also evaluated the proportion of translocated photo-assimilates under both normal and bleached conditions. We also evaluated the acclimatization capacity of the endolithic community by studying their pigments’ composition and their temporal changes during short-term incubations.

Our result shows that a similar or higher amount of organic carbon was translocated in healthy corals when compared with bleached corals. Similarly, we found comparable photosynthetic performances of endoliths under healthy and bleached coral conditions during the illuminated period of our incubations. This showed that photosynthetic performance of endolithic algae was not affected by high illumination under the bleached coral tissues, in spite of some decrease in the concentrations of chlorophyll (Chl) *a*. This means that the endolithic community can still support the coral holobiont for some period of time, even under stressful conditions.

## 2. Material and methods

### 2.1. Coral sampling

Observations were made in May 2016 at Sesoko coral reef lagoon located on the western side of Sesoko Island, Okinawa, Japan (26°39′ N, 127°51′ E). Live coral coverage was approximately 8% and was dominated by non-branching corals (mainly *Porites* spp., *Goniastrea* spp. and *Favia* spp.) [21]. Fragments of healthy colonies of *Porites lutea* were sampled with permission from the Okinawa Prefectural Government, No. 28-6, for incubation experiments and identification of the endolithic community. Sampling was performed during low tide (depth 0.5–1 m). Environmental conditions at the time of sampling were registered using a multi-sensor Hydrolab MS5 Multiparameter Sonde (OTT Hydromet, Kempten, Germany). The temperature and salinity of the seawater were 27.5°C and 34 ppt, respectively. Coral samples were immediately transported to the laboratory and kept in an aquarium with running natural seawater.

### 2.2. Taxonomic identification of endolithic algae

The soft tissue layer of corals was separated from the skeleton using a handsaw. The coral skeleton containing the green band layer formed by the endolithic community was decalcified in 1 N HCl until complete dissolution, and the emerging endolithic algae were fixed in formaldehyde-buffered solution (4% final concentration). Aliquots of endolithic algae were mounted on glass slides and observed at 4×, 20×, 40× and 100× magnification under a light microscope (Nikon-ECLIPSE/80i). Algae were identified using key morphological characteristics as described in taxonomic references [14,15,22–24].

### 2.3. Incubation design

A natural isotope tracer technique using ^13^C and ^15^N was applied to measure the primary production and nitrogen fixation rates. The following two incubation experiments were performed by Addition: corals were incubated in seawater with addition of ^13^C and ^15^N gas to assess the primary production and N_2_ fixation in the coral tissue and the endolithic community; and Injection: corals were incubated in the same manner but ^13^C solution was directly injected into the endolithic green band. Healthy and partially bleached corals were subjected to these two incubation experiments. For this, fragments of the same coral colonies were divided into two parts: healthy coral fragments were acclimatized for 3 days under ambient conditions with running seawater, and the remaining coral fragments were induced to bleach at a seawater temperature of 33°C under dark conditions.

In the Addition incubation, three replicates of healthy and partially bleached coral fragments of approximately the same size were placed into individual polycarbonate Nalgene bottles (1230 ml). The incubation bottles were filled with seawater collected at the site of coral collection. The ^13^C tracer solution (2.46 ml; NaH^13^CO_3_, 99.9% ^13^C), prepared by adding 1 g of the salt to 100 ml deionized water, was added to the incubation bottle to achieve a final ^13^C concentration of approximately 11.5% (a ^13^C enrichment factor of 10.4). Subsequently, 2.46 ml of ^15^N_2_ gas (99.8% ^15^N, Shoko Co. Ltd, Tokyo, Japan) was added using a gas-tight syringe to obtain an enrichment of 6.8% in the seawater. The incubation bottles containing the coral fragments were set up *in situ* at a depth of approximately 5 m, with an average illumination of 360 µmol photons m^−2^ s^−1^ and an average temperature of 26°C. The incubations were performed from 06.00 to 18.00 h (12 h light period) and from 06.00 to 06.00 h on the next day (24 h incubation). The water temperature and light intensity during the incubations were monitored using *in situ* sensors (MDS-MkV/T and MDS-Mk/L, Alec Electronics, Kobe, Japan).

In the Injection incubation, the ^13^C solution was added directly to the green band using a syringe after opening a narrow hole (less than 2 mm in diameter) inside the coral skeleton that was sealed after the Injection. Three replicates each for the 12 h illumination and 24 h (12 L : 12 D) incubation were set up in an indoor incubation system on the premises of the Tropical Biosphere Research Center of the University of Ryukyus, Sesoko Island, Okinawa. The incubation was performed at 27°C and 300 or 0 μmol photons m^−2^ s^−1^ light intensity.

### 2.4. Treatment of samples

After incubation, the coral fragments were separated into coral soft tissue and the endolithic green band layer. The latter was ground using a pestle and mortar and homogenized; the homogenate was acidified with 1 N HCl to remove carbonates, filtered through a pre-combusted (500°C) Whatman GF/F filter (47 mm), and finally, stored at −20°C until subsequent treatments. Samples on the filters were dried at 60°C in an electric oven until they attained constant weight. Particulate organic carbon (POC), particulate organic nitrogen (PON), and ^13^C and ^15^N content were measured using a mass spectrometer (DELTA plus Advantage, Thermo Finnigan Co., USA, equipped with an elemental analyser EA1110). The primary production was calculated according to Hama *et al*. [25] and N_2_ fixation was calculated using a modified method as described by Casareto *et al*. [26], which was based on the method of Montoya *et al*. [27].

### 2.5. Maximum quantum yield (Fv/Fm) of endolithic algae and symbiotic zooxanthellae

Chlorophyll *a* fluorescence was measured at the start and after 12 h and 24 h of incubations using a pulse-amplitude-modulated (PAM) fluorometer (JUNIOR PAM, Walz, Germany) according to the method of Schreiber *et al*. [28]. The maximum quantum yield was calculated as Fv*/*Fm, where Fv = Fm − Fo. Here, Fo is the initial fluorescence after dark adaptation and Fm is the maximum fluorescence after dark adaptation [29]. Coral samples were kept for 15–30 min in the dark for adaptation before the fluorescence measurements were obtained. The fluorescence data were registered from three to six different points in both coral tissues and the endolithic green band, from which averages and standard errors were calculated.

### 2.6. Pigment composition

Pigments were analysed at the start and after 12 h and 24 h of incubation in coral tissues, and the endolithic green band subsampled from the incubated corals. Measurements were obtained using high-performance liquid chromatography (HPLC) (LC-30AD; Shimadzu, Kyoto, Japan). The HPLC system was equipped with a ZORBAX Eclipse Plus C8 Column (2.1 × 50 mm; Agilent Technology, Santa Clara, CA, USA). The subsamples of coral tissues and endolithic algae were ground in a mortar, filtered through a GF/F filter and preserved at −20°C until the measurements were obtained. The pigments were extracted with 2 ml of *N*,*N*-dimethylformamide and maintained overnight in the dark at −20°C. The extract was filtered through a syringe filter (0.2 µm, Millex-LG; Millipore, Bedford, MA, USA) to remove cell debris. The pigment extract (1 ml) was mixed with 0.2 ml of ultrapure water (Milli-Q, Millipore) and each 10 µl of the mixed solution was injected into the HPLC system using an autosampler (SIL-30AC, Shimadzu). The pigments were eluted at a flow rate of 0.3 ml min^−1^ at 25°C using a programmed binary gradient. All samples were prepared under subdued light. The solvents used were as follows: (A) methanol : acetonitrile : 0.25 M aqueous pyridine solution (50 : 25 : 25, vol : vol : vol); (B) methanol : acetonitrile : acetone (20 : 60 : 20, vol : vol : vol). The separated pigments were detected spectrophotometrically using a photodiode array detector (SPD-M30AD; Shimadzu) with an optical resolution of 1.2 nm over a wavelength range from 320 to 720 nm. Each peak was identified by comparing HPLC retention times with the absorption spectra of standards and the data obtained from photodiode array detection. The photosynthetic pigment concentrations in the coral tissue and endolithic algae were normalized to the coral surface area (square centimetre).

### 2.7. Statistical analysis

MINITAB v. 14 software was used for statistical analysis. ANOVA was performed to determine the significant differences among factors and Tukey's *post hoc* test was used to assess the pairwise differences when ANOVA revealed statistically significant effects (*p* < 0.01 and *p *< 0.05).

## 3. Results

### 3.1. Composition of the endolithic community

**Figure 1.**
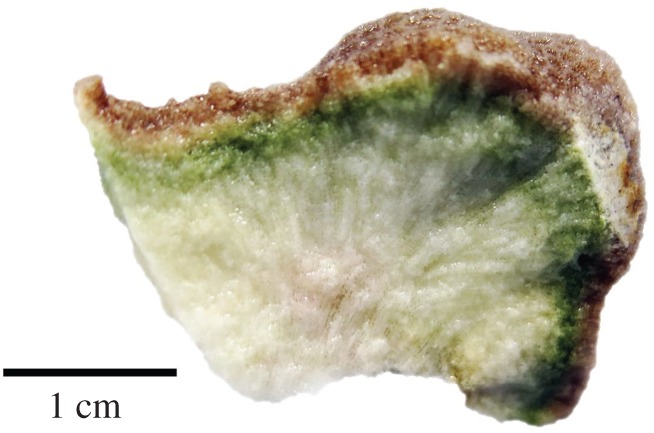
Distinct green layer formed by the endolithic community in the skeleton of *Porites lutea*.

**Figure 2.**
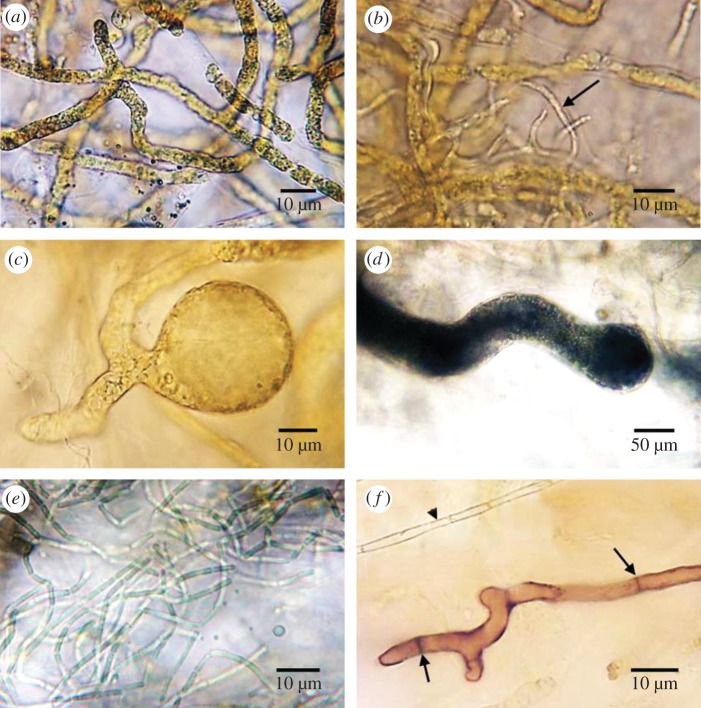
Light-microscope photographs of the endolithic community from the green layer in the skeleton of *Porites lutea*. *Ostreobium quekettii* ((*a*) main branches, (*b*) ultimate branches (arrow) and (*c*) swelling similar to sporangia), (*d*) holdfast of dasycladalean alga, (*e*) *Leptolyngbya terebrans*. (*f*) Fungi: hyaline hypha (arrow head), red hypha with septum (arrows).

The endolithic community forming a green band in the coral skeleton underneath the coral soft tissue is shown in a cross section of *P. lutea* (figure 1). Two groups of algae (two species of Chlorophyta and Cyanophyta) and a fungus dominated the community. Among the Chlorophyta, *Ostreobium quekettii* was largely dominant, showing main branches (3–10 µm in diameter; figure 2*a*), ultimate branches (0.5–1.5 µm in diameter; figure 2*b*) and swellings similar to sporangia (approx. 20–300 µm in diameter; figure 2*c*). Dasycladales (holdfast) were present in low abundance, showing irregular branches (30–100 µm in diameter; figure 2*d*). The cyanobacterium *Leptolyngbya terebrans* was present in low abundance, showing non-heterocystous filaments (0.8–1.3 µm in diameter; figure 2*e*). Two fungal forms were found in low abundance with red and hyaline thallus, 2–5 µm and 1–2 µm in diameter, respectively (figure 2*f*).

### 3.2. Photosynthetic activity

**Figure 3.**
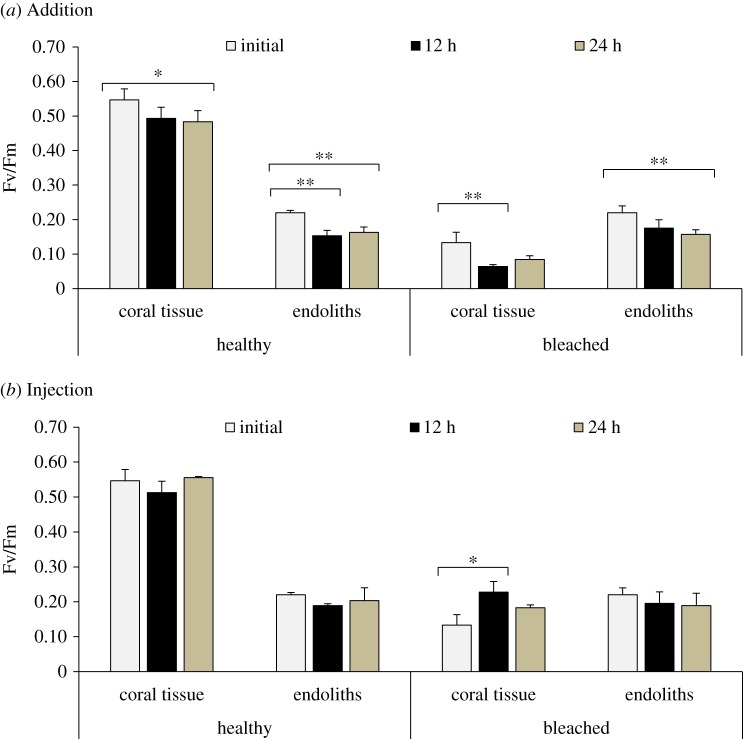
Maximum quantum yield (Fv/Fm, average ± standard error) of the coral tissues and endolithic algae in healthy and bleached corals (*n* = 3) measured during Addition incubation (*a*) and Injection incubation (*b*). Three-way ANOVA and *post hoc* Tukey's test were used to determine significant differences among the conditions (healthy or bleached), layers and times. * and ** indicate significant differences at *p < *0.05 and *p < *0.01, respectively.

The maximum photosynthetic quantum yields (Fv/Fm) of zooxanthellae in coral tissues and endolithic algae in both healthy and bleached colonies are shown in figure 3. The Fv/Fm in the healthy coral tissues was almost stable or decreased slightly after 24 h during Addition incubation; however, in bleached coral tissues, the initial value of Fv/Fm was significantly lower than that of healthy corals and varied significantly (*p *< 0.01 and *p *< 0.05) after exposure to light for 12 h in both the Addition and Injection incubations. The initial Fv/Fm values for the endolithic algae (Addition incubation) in healthy and bleached corals were similar, and after 12 and 24 h, the Fv/Fm values differed significantly (*p *< 0.01) from the initial values. In healthy corals, the Fv/Fm of the endoliths significantly decreased (*p* < 0.01) after 12 and 24 h, showing a slight recovery; however, in bleached corals, the Fv/Fm of endoliths showed a continuous decrease after 12 and 24 h (*p *< 0.01). Similar patterns were observed during Injection incubation, but no significant differences were observed. In spite of that, overall the Fv/Fm values of the endoliths after the incubations showed similar values in healthy and bleached corals, indicating comparable photosynthetic performance in healthy and bleached conditions.

### 3.3. Photosynthetic pigments and changes in their composition during the incubations

The pigment concentrations in the coral tissues and endolithic algae before the start of the incubations are shown in table 1. Pigments identified included Chl *a*, *b*, *C*_2_, 13^2^,17^3^- cyclopheophorbide *a* enol (cPPB-*a*E), and carotenoids (peridinin; tracer pigment of dinoflagellate, diadinoxanthin and *β* carotene), which were identified together with zeaxanthin (tracer pigment of cyanophytes). Moreover, Chl *a* spectra could be separated into three peaks by HPLC elution profiles: Chl *a* allomer, Chl *a* and Chl *a* epimer [30]. From these data, the Chl *a *: carotenoids, Chl *a* : Chl *b* and allomer : total Chl *a* ratios were calculated.

**Table 1.** Pigment concentrations in the coral tissues and endolithic algae of *Porites lutea* before incubation (µg cm^−2^: mean ± s.e.; *n* = 3). One-way ANOVA and *post hoc* Tukey's test were used to determine significant differences between the different conditions (healthy or bleached). ** indicates significant differences at *p* < 0.01. Unit: µg cm^−2.^

pigments	healthy	bleached
*coral tissue*		
chlorophyll *a* spp.	13.59 ± 0.97	2.11 ± 0.25**
chlorophyll *c*_2_	0.26 ± 0.05	0.38 ± 0.08
cPPB-*a*E	0.51 ± 0.13	0.75 ± 0.12
peridinin	5.93 ± 0.39	2.18 ± 0.10**
diadinoxanthin	2.35 ± 0.04	1.86 ± 0.09**
β-carotene	0.25 ± 0.04	0.25 ± 0.04
zeaxanthin^a^	0.98 ± 0.24	1.08 ± 0.15
lutein^a^	0.40 ± 0.15	0.74 ± 0.39
chlorophyll *a* : carotenoids^b^	1.61 ± 0.13	0.49 ± 0.06**
allomer : total Chl *a*	0.30 ± 0.06	0.35 ± 0.02
*endolithic algae*		
chlorophyll *a* spp.	2.96 ± 0.13	2.27 ± 0.05
chlorophyll *b* spp.	1.26 ± 0.04	0.69 ± 0.04
zeaxanthin	0.04 ± 0.00	0.04 ± 0.00
β-carotene	1.38 ± 0.00	0.34 ± 0.02**
chlorophyll *a* : chlorophyll *b*	2.38 ± 0.13	2.96 ± 0.14
chlorophyll *a* : carotenoids	0.97 ± 0.09	2.50 ± 0.32**
allomer : total Chl *a*	0.22 ± 0.15	0.23 ± 0.05

^a^Pigment of associated algae (other than zooxanthellae) with coral tissue.

^b^Carotenoids found in zooxanthellae.

In the healthy coral tissues, Chl *a*, peridinin and diadinoxanthin concentrations were significantly (*p* < 0.01) higher than in the bleached corals and the Chl *a *: carotenoids ratio in the bleached corals was significantly (*p* < 0.01) lower than that in the healthy corals. Allomer : total Chl *a* was slightly higher in bleached corals but did not show a significant difference.

**Figure 4.**
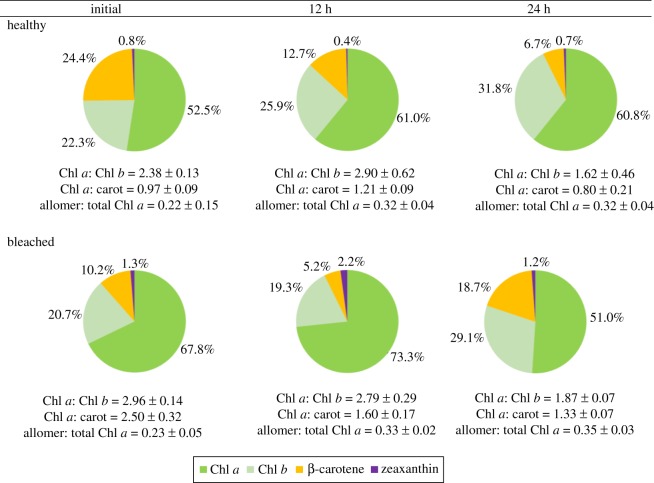
Percentage composition of chlorophylls and carotenoids in the endolithic algae of healthy and bleached corals at initial measurement, 12 h (light period) and 24 h (light and dark periods).

In the endolithic green band, the concentrations of Chl *a*, *b* and the Chl *a* *:* Chl *b* ratio did not show a significant difference between healthy and bleached corals. The Chl *a *: carotenoids ratio was significantly lower in the green bands of the healthy corals than in those of the bleached corals. Allomer : total Chl *a* was slightly higher in bleached corals but did not show a significant difference. Allomer : total Chl *a* was higher in coral tissue than in endoliths, denoting a more oxidative state of Chl *a* in coral tissues. The concentration of zeaxanthin was higher in coral tissues, denoting an important association with cyanobacteria. Changes in the pigment percentage of endoliths during the incubations are shown in figure 4 and electronic supplementary material, table S1. The percentage of Chl *a* in endoliths of bleached corals was higher than that in healthy corals and increased during 12 h of incubation from 68 to 73% (bleached) and 52 to 61% (healthy). However, after 24 h of incubation, the values decreased in both coral conditions. The percentage of Chl *b* in endoliths of healthy corals was higher than that in the bleached corals and increased along incubations in both coral conditions. From these results, the percentage of Chl *a* decreased with an important increase in Chl *b* and carotenoids, and this resulted in a drastic decrease in the Chl *a* : Chl *b* and Chl *a *: carotenoid ratios.

### 3.4. Primary production and nitrogen fixation

The results for primary production and N_2_ fixation are shown in table 2, and the changes in POC, PON and the POC : PON ratio, measured during the two incubations in healthy and bleached *P. lutea*, are shown in electronic supplementary material, table S2. During the Addition incubation, primary production in the healthy coral tissues reached a value of approximately 40 µg C cm^−2^ during the illuminated hours and decreased slightly after 24 h due to respiration during the night. However, in the bleached coral tissues, primary production during the illumination period was lower than 1 µg C cm^−2^ and a slight increase (2.5 µg C cm^−2^) was observed after 24 h of incubation, which may indicate some external source of fixed organic matter. By contrast, the endolithic algae in the healthy corals showed a very low (0.23 µg C cm^−2^) primary production rate during the 12 h light period, and the rate increased after 24 h of incubation. The primary production rate of the endolithic algae in the bleached corals decreased slightly compared to the production in the healthy corals and decreased after 24 h of incubation, revealing higher respiration than in the endoliths of healthy corals.

**Table 2.** Primary production and N_2_ fixation in the coral tissues and endolithic algae of healthy and bleached corals (*n = 3*) after 12 h (light period) and 24 h (light and dark periods) measured during Addition and Injection incubations.

			primary production (µg C cm^−2^ time^−1^)		N_2_ fixation (ng N cm^−2^ time^−1^)	
incubation	condition	layer	12 h	24 h	12 h	24 h
Addition	healthy	tissue	39.88 ± 0.6	35.23 ± 2.2	29.61 ± 9.3	13.41 ± 3.1
		endoliths	0.23 ± 0.0	0.54 ± 0.0	2.12 ± 0.1	1.77 ± 0.5
	bleached	tissue	0.64 ± 0.0	2.51 ± 0.0	7.66 ± 0.7	12.38 ± 2.0
		endoliths	0.16 ± 0.0	0.11 ± 0.0	1.59 ± 0.2	1.32 ± 0.1
Injection	healthy	tissue	69.67 ± 4.6	65.79 ± 0.3		
		endoliths	2.18 ± 0.7	6.24 ± 0.7		
	bleached	tissue	8.73 ± 0.0	22.94 ± 1.3		
		endoliths	3.45 ± 0.0	3.16 ± 0.1		

During the Injection incubation, the trend of primary production in healthy and bleached coral tissues was consistent with the results obtained during the Addition incubation. However, primary production values during the 12 h light period were approximately two times higher for the healthy coral tissues (70 µg C cm^−2^) and one order of magnitude higher (approx. 9 µg C cm^−2^) for the bleached coral tissues when compared to the values obtained during the Addition incubation. The values for primary production in the endolithic algae were also higher than those obtained during the Addition incubation. Approximately, three times more carbon was fixed by the endolithic algae present in the healthy corals after 24 h of incubation. As in the Addition incubation, consumption (respiration) of organic carbon by endolithic algae in the bleached corals was observed after 24 h of incubation.

Nitrogen fixation was detected in both coral tissues and the endolithic community. In healthy coral tissues, approximately 30 ng N cm^−2^ was fixed during the 12 h light period and more than 50% was consumed during the night after 24 h. However, in the tissues of the bleached corals, N_2_ fixation was lower (around 8 ng N cm^−2^) during the 12 h light period and increased by 1.6-fold during the night period after 24 h of incubation. Nitrogen fixation in the endolithic community was slightly lower (approx. 25%) in the bleached corals (1.6 ng N cm^−2^) when compared to that in the healthy corals (2.12 ng N cm^−2^). In both healthy and bleached conditions, N_2_ fixation of endoliths decreased after 24 h. This pattern may also indicate some consumption and/or translocation of N-rich organic matter in the coral tissue. In coral tissues, the POC : PON ratio (electronic supplementary material, table S2) increased after 12 h and 24 h of incubation, indicating an increase in the C-rich organic matter. By contrast, the POC : PON ratio in the endolithic algae increased during the illuminated period (12 h) and decreased slightly or remained the same after the 24 h incubation.

### 3.5. Translocation

**Figure 5.**
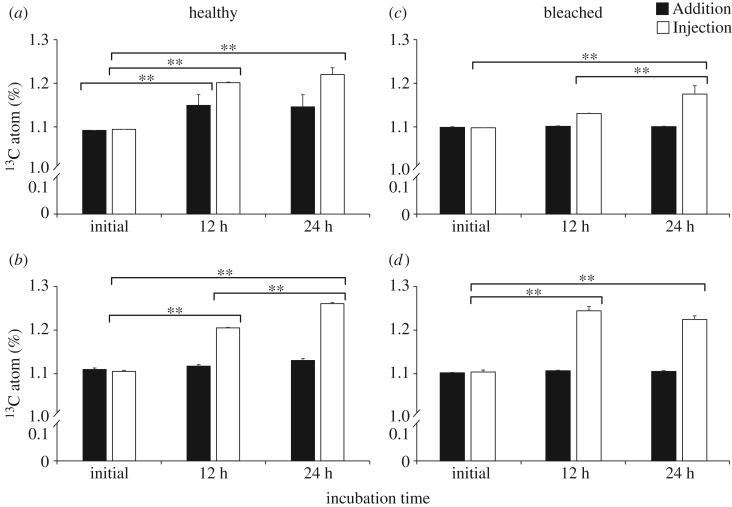
^13^C atom (%) recorded in *Porites lutea* (*n* = 3) at initial measurement, 12 h (light period) and 24 h (light and dark periods). (*a,b*) Healthy corals: (*a*) coral tissue and (*b*) endolithic algae. (*c,d*) Bleached corals: (*c*) coral tissue and (*d*) endolithic algae. Two-way ANOVA and *post hoc* Tukey's test were used to determine significant differences among the different incubations and times. * and ** indicate significant differences at *p *< 0.05 and *p *< 0.01, respectively.

Incorporation of ^13^C atoms (%) in the coral soft tissues and in the endolithic community during Addition and Injection incubations for both healthy and bleached corals is shown in figure 5 and electronic supplementary material, table S3. During Addition incubation, incorporation of ^13^C atoms (%) in healthy coral tissues increased (5.5%) after 12 h of incubation (*p *< 0.01) and slightly decreased to 4.5% after 24 h. However, during the Injection incubation, the incorporation of ^13^C atoms (%) was higher, and it increased by 8.8% during the 12 h light period (*p *< 0.01) and by 12.4% after the 24 h incubation period (*p *< 0.01). In the endolithic community of healthy corals, the incorporation of ^13^C atoms (%) was almost undetectable during Addition incubation; however, during Injection incubation it was highly increased (7.1%) (*p *< 0.01) during the first 12 h and increased by 14.3% (*p *< 0.01) after 24 h. In the bleached coral tissue, ^13^C atoms (%) were almost not incorporated (less than 1.0%) throughout the Addition incubation; however, an increase of 2.1% after 12 h and of 6.9% (*p *< 0.01) after 24 h was observed during the Injection incubation. As in the case of healthy corals, incorporation of ^13^C atoms (%) in the endolithic algae of bleached corals was not detected during the Addition incubation. However, it was high during the first 12 h of Injection incubation, with an increase of 11.5% (*p *< 0.01), and this result was consistent with the primary production rates. Some of the incorporated ^13^C atoms (%) (2.1%) (*p *< 0.01) were lost (respired and/or used) after the 24 h incubation.

## 4. Discussion

### 4.1. Coral acclimatization to stressful conditions

Associated microorganisms in the coral holobiont show the capacity to rapidly acclimatize to environmental changes by modifying their population growth and physiological responses. During our incubations, the pigment concentrations in coral tissues showed a pattern of rapid acclimatization to the changing environment (table 1; electronic supplementary material, table S1). The proportion of Chl *a* in the healthy coral tissues was much higher than that in the bleached corals, and during our incubations, the proportion of carotenoids increased in the bleached corals with a concomitant reduction of the Chl *a *: carotenoid ratio. The photoprotective and antioxidant functions of carotenoids, particularly their importance in the xanthophyll cycle and *β* carotene variations, are well known [31–33]. In our study, the decrease in the Chl *a *: carotenoid ratio during 12 h light incubation of bleached corals resulted from extensive decrease in Chl *a* relative to carotenoids, showing how zooxanthellae respond to stressful conditions.

### 4.2. Photosynthetic pigments of endolithic community

The endolithic community that lives under the coral tissue layer is adapted to photosynthesis in the dark environment shaded by the zooxanthellae living inside the coral tissues [34]. In this study, *O. quekettii* largely dominated the endolithic photosynthetic community of *P. lutea*. Other studies have also found *Ostreobium* spp. as the dominant endolithic algae in living and dead corals [35,36]. In particular, *Ostreobium* spp. are known for their capacity to harvest light under low light intensity because of their exclusive photosynthetic pigment composition, which includes Chl *b* and far red-absorbing Chl *a* that act as antennae under lower illumination conditions [37]. The Chl *a *: Chl *b* ratio in plants is widely used as an indicator of tolerance in shaded areas [38]. The normal shade-adapted ratio in higher plants is approximately 3, whereas highly shade-adapted *Ostreobium* spp. may reach a ratio of 1.26–2.8 [35,39]. In this study, the Chl *a *: Chl *b* ratio of endoliths varied from 1.6 to 2.9 (healthy corals) and 1.9 to 3 (bleached corals) (electronic supplementary material, table S1 and figure 4). Figure 4 shows that, in the endoliths of healthy corals, after 12 h light and 24 h light plus dark incubations, the proportion of Chl *b* increased, and the Chl *a* : Chl *b* ratio decreased after 24 h. By contrast, Chl *b* decreased and Chl *a* increased during the 12 h light incubations in the endoliths of bleached corals, showing a rapid response to the better illumination condition under the bleached coral tissues. The increased proportion of Chl *a* in the bleached condition resulted in a higher Chl *a* : carotenoid ratio. However, after 24 h of incubation, the drastic drop in this ratio shows that the endolithic algae might be affected to some extent by oxidative stress after being exposed to higher illumination. This pattern can be also observed from the increase in allomer : total Chl *a* along the incubation, as this ratio can be used to estimate the oxidative state of Chl *a* [40]. These rapid variations in the pigments of endolithic algae illustrate their plasticity to acclimatize to drastic environmental changes. Moreover, our measurements of photosynthetic quantum yield (Fv/Fm) of endolithic algae living in healthy and bleached corals (figure 3) showed that Chl *a* fluorescence measurement of the endolithic community was similar in these two conditions. This result reveals comparable photosynthetic performance under both coral conditions, which is in agreement with previous observations [41]. Nevertheless, a slight decrease in Chl *a* fluorescence measurement after 24 h of incubation showed that some stress under non-shading conditions might decrease the photosynthetic performance with time. This result is in agreement with the important decrease in the Chl *a* : carotenoid ratio.

### 4.3. Carbon fixed by endoliths

The endolithic community has been proposed to assist in coral survival when the coral is bleached [18]; however, there are no available data on the amount of organic matter (in terms of carbon and nitrogen) synthesized by the endolithic community. The most notable result in this study is the quantitative measurement (in terms of organic C and N) of primary production, nitrogen fixation and translocation from the endolithic community. These measurements were obtained under both normal and bleached conditions. Our incubation design allowed us to measure the primary production of zooxanthellae in coral tissues (Addition incubation) and that in the endolithic community (Injection incubation). Moreover, we calculated the proportion of carbon photo-assimilates that the endolithic community could transfer to the coral tissues under both healthy and bleached conditions. In the Addition incubation, ^13^C was added to the water in incubation bottles containing coral pieces; therefore, uptake of ^13^C by zooxanthellae and their primary production rates were well measured. However, ^13^C did not reach the endolithic zone in a direct and timely manner, and therefore, the primary production of endoliths was highly underestimated. In the Injection incubation, ^13^C solution was directly added inside the green band of the endoliths; therefore, primary production of the endolithic algae was clearly revealed (table 2) and the translocation could be estimated by following the increase in ^13^C atoms (%) in coral tissues (figure 5). The difference in ^13^C atom content (%) between the Injection and Addition incubations allowed us to calculate the proportion of translocated assimilates from endoliths.

Our incubations revealed that translocation of organic matter occurred in both healthy and bleached conditions. Moreover, our data show that translocation in the healthy condition was higher than that in the bleached condition; this pattern differs from the results of a previous study by Fine & Loya [18]. Even if the endolithic community also showed some stress signals under the bleached coral condition, the Chl *a* fluorescence data showed that the photosynthetic performance was comparable between the two conditions; therefore, the endolithic community was also able to support the coral host under stressful conditions.

Another important feature revealed by the Addition incubation is that endoliths in the healthy corals may fix carbon over a longer period of time owing to the increase in their primary production during the dark hours. This is due to their capacity to harvest light even under low illumination conditions [34], and therefore might surpass our incubation time set-up of 12 h light. Moreover, the primary production of endoliths in the bleached corals was lower than that of endoliths in healthy corals. This pattern is in agreement with the lower Chl *a* concentration (electronic supplementary material, table S1) and can be interpreted as a high respiration rate of endoliths in the bleached corals owing to oxidative stress. This can be attributed to the effect of high illumination resulting in inhibition of the photosystem of the algae because they are more adapted to photosynthesize under low light conditions. The lower primary production of endoliths in bleached corals (0.11 µg C cm^−2^ d^−1^ under the bleached condition versus 0.54 µg C cm^−2^ d^−1^ in the healthy condition) and the concomitant increase of primary production in coral tissues (0.64–2.51 µg C cm^−2^ d^−1^) exemplify the translocation pattern from the endoliths towards the bleached coral tissues during the dark period. However, ^13^C atom (%) incorporation showed that translocation in healthy corals was higher, meaning that corals in the healthy condition may have higher respiration (consumption) rates. Variations in the POC : PON ratio over the period of incubation (electronic supplementary material, table S2) also showed that the ratio increased along the incubations in the coral tissues, revealing an increase in C-rich organic matter.

### 4.4. Nitrogen fixed by endoliths

N_2_ fixation is an important function to take into account when considering the entire metabolism of the holobiont. Our measurements of N_2_ fixation during the Addition incubation revealed that this process occurred in both the coral tissues and endolithic band. Cyanobacteria are known as major diazotrophs (nitrogen-fixing organisms) together with other bacteria such as the alpha-proteobacteria. In our study, the presence of zeaxanthin, a tracer pigment for cyanobacteria [42], in the coral tissue and in the endolithic green band confirms the presence of cyanobacteria at both sites. Lesser *et al*. [7] also described the presence of N_2_ fixer unicellular cyanobacteria in the tissue of *Montastraea cavernosa*. In the endolithic band, we found the presence of the cyanobacterium, *L. terebrans*. *Plectonema* spp. (synonym of *Leptolyngby*a) are known to fix nitrogen at low oxygen levels, and they are not able to fix it in the anoxic environment, similar to most diazotrophs [43]. In previous genetic studies on microbes associated with *P. lutea* collected from the same sampling area, nitrogen-fixing cyanobacteria and alpha-proteobacteria were identified in both the coral tissue and coral skeleton [44].

Our measurements showed that N_2_ fixation rates in the coral tissues were four times higher in the healthy corals than in the bleached corals. However, the N_2_ fixation rates of the endoliths in the healthy and bleached corals were similar. Moreover, N_2_ fixation was higher during the first 12 h of the illuminated period, except in the case of bleached corals in which N_2_ fixation was increased, and this pattern was similar to that of primary production. This result suggests that some nitrogenous compounds can be also translocated from the endolithic layer towards the coral tissue.

## 5. Conclusion

Overall, based on the results of this study, we can conclude that the endolithic community plays a crucial role in supporting the coral holobiont under normal as well as stressful conditions. Rapid responses by changing their pigments to different illumination conditions and extended photosynthetic period are important acclimatization features of the endolithic community to rapid environmental changes. Not only under stressful but also under normal conditions, photo-assimilates from the endoliths are translocated to the coral tissues together with some nitrogen-rich compounds. Our study is the first to provide quantification of the primary production, N_2_ fixation and translocation of organic matter from the endolithic community towards the coral tissues in both the normal and bleached coral scenarios. Further studies are necessary to understand how photo-assimilates are transported during translocation. Moreover, photoprotection by the production of antioxidants in the endolithic community needs to be investigated.

## Supplementary Material

Table S1

## Supplementary Material

Table S2

## Supplementary Material

Table S3
