# Supplementary material for: 13C and 15N assimilation and organic matter translocation by the endolithic community in the massive coral Porites lutea
Source: R Soc Open Sci. 2017 Dec 6;4(12):171201. doi: 10.1098/rsos.171201 (PMC5750018; doi:10.1098/rsos.171201)
Supplement: Table S1 [file rsos171201supp1.pdf]

Table S1. Pigment concentration (µg cm^{-2}) in the coral tissues and in the endolithic algae measured during incubations at initial, 12 h (light period), and 24 h. 12 h = light period, 24 h = light and dark periods

pigment	healthy			bleached		
	**initial**	**12 h**	**24 h**	**initial**	**12 h**	**24 h**
**coral tissue**						
Chlorophyll *a* spp.	13.59 ± 0.97	11.01 ± 0.24	13.47 ± 0.24	2.11 ± 0.25	0.55 ± 0.14	0.87 ± 0.05
Chlorophyll c_2	0.26 ± 0.05	0.15 ± 0.01	0.14 ± 0.01	0.38 ± 0.08	0.08 ± 0.02	0.07 ± 0.00
cPPB-*a*E	0.51 ± 0.13	0.67 ± 0.18	0.44 ± 0.10	0.75 ± 0.12	1.17 ± 0.24	0.66 ± 0.02
Peridinin	5.93 ± 0.39	3.85 ± 0.09	5.34 ± 0.45	2.18 ± 0.10	0.77 ± 0.06	1.02 ± 0.06
Diadinoxanthin	2.35 ± 0.04	1.11 ± 0.13	0.65 ± 0.03	1.86 ± 0.09	0.55 ± 0.02	0.57 ± 0.03
β-carotene	0.25 ± 0.04	0.19 ± 0.04	0.34 ± 0.07	0.25 ± 0.04	0.15 ± 0.06	0.28 ± 0.07
[1] Zeaxanthin	0.98 ± 0.24	0.96 ± 0.20	0.58 ± 0.14	1.08 ± 0.15	0.14 ± 0.06	0.30 ± 0.12
[1] Lutein	0.40 ± 0.15	0.58 ± 0.27	0.28 ± 0.11	0.74 ± 0.39	0.06 ± 0.02	0.23 ± 0.11
[2] Chlorophyll *a* : diatoxanthin	4.44 ± 1.04	1.75 ± 0.45	1.85 ± 0.79	2.60 ± 0.68	1.11 ± 0.18	1.18 ± 0.49
[3] Chlorophyll *a* : Carotenoids	1.61 ± 0.13	2.15 ± 0.08	2.19 ± 0.17	0.49 ± 0.06	0.36 ± 0.09	0.46 ± 0.02
allomer : total Chl *a*	0.30 ± 0.06	0.42 ± 0.07	0.36 ± 0.10	0.35 ± 0.02	0.53 ± 0.08	0.43 ± 0.04
**endolithic algae**						
Chlorophyll *a* spp.	2.96 ± 0.13	2.73 ± 0.16	3.04 ± 0.05	2.27 ± 0.05	1.12 ± 0.23	1.62 ± 0.04
Chlorophyll *b* spp.	1.26 ± 0.04	1.16 ± 0.20	1.59 ± 0.18	0.69 ± 0.04	0.30 ± 0.06	0.92 ± 0.04
Zeaxanthin	0.04 ± 0.00	0.02 ± 0.00	0.03 ± 0.00	0.04 ± 0.00	0.03 ± 0.01	0.04 ± 0.00
β-carotene	1.38 ± 0.00	0.57 ± 0.18	0.33 ± 0.22	0.34 ± 0.02	0.08 ± 0.03	0.63 ± 0.06
[2] Chlorophyll *a* : Siphonein	4.39 ± 1.02	4.68 ± 1.52	7.65 ± 2.62	4.26 ± 1.05	7.20 ± 2.71	3.00 ± 0.37
[2] Chlorophyll *a* : Neoxanthin	8.34 ± 3.57	12.27 ± 2.25	7.90 ± 4.80	14.64 ± 2.62	9.77 ± 3.33	7.94 ± 2.03
Chlorophyll *a* : Chlorophyll *b*	2.38 ± 0.13	2.90 ± 0.62	1.62 ± 0.46	2.96 ± 0.14	2.79 ± 0.29	1.87 ± 0.07
Chlorophyll *a* : Carotenoids	0.97 ± 0.09	1.21 ± 0.09	0.80 ± 0.21	2.50 ± 0.32	1.60 ± 0.17	1.33 ± 0.07
allomer : total Chl *a*	0.22 ± 0.15	0.33 ± 0.04	0.35 ± 0.03	0.23 ± 0.05	0.33 ± 0.02	0.35 ± 0.03

Unit: µg cm^{-2}

1 Pigment of associated algae (other than zooxanthellae) with coral tissue

2 Ratio calculated from peak area of HPLC elution profiles

3 Carotenoids found in zooxanthellae

Table S1: Sangsawang et al., 2017
