# Supplementary material for: 13C and 15N assimilation and organic matter translocation by the endolithic community in the massive coral Porites lutea
Source: R Soc Open Sci. 2017 Dec 6;4(12):171201. doi: 10.1098/rsos.171201 (PMC5750018; doi:10.1098/rsos.171201)
Supplement: Table S2 [file rsos171201supp2.pdf]

Table S2. POC and PON (mg cm^{-2} ± standard error) in the healthy and bleached *Porites lutea* (coral tissues and endolithic community) during the two incubations at initial, 12 h (light period), and 24 h (light and dark periods).

incubation	condition	layer	POC			PON			POC: PON		
			**initial**	**12 h**	**24 h**	**initial**	**12 h**	**24 h**	**initial**	**12 h**	**24 h**
**Addition**	healthy	tissue	5.81±0.85	8.13±6.84	8.20±3.29	0.71±0.07	0.81±0.41	0.62±0.05	8.11±0.43	9.07±1.74	13.01±2.77
		endoliths	0.80±0.28	0.46±0.05	0.35±0.11	0.06±0.00	0.03±0.00	0.03±0.01	13.87±3.99	13.93±1.30	10.39±0.90
	bleached	tissue	4.84±0.55	2.26±0.06	3.66±0.54	0.49±0.00	0.25±0.01	0.37±0.09	9.79±1.17	9.16±0.30	10.11±0.87
		endoliths	0.37±0.07	0.41±0.11	0.32±0.01	0.05±0.01	0.04±0.01	0.03±0.01	7.24±0.42	10.96±0.60	9.70±1.60
**Injection**	healthy	tissue	5.30±0.06	6.61±0.21	4.56±1.31	0.76±0.01	0.89±0.08	0.58±0.14	6.98±0.02	7.46±0.51	7.81±0.38
		endoliths	1.01±0.04	0.27±0.11	0.39±0.02	0.14±0.01	0.03±0.01	0.05±0.01	7.19±0.16	8.60±0.66	8.51±0.32
	bleached	tissue	4.72±0.10	2.97±0.63	4.63±1.72	0.71±0.02	0.24±0.02	0.53±0.16	6.68±0.06	12.15±1.16	8.55±0.43
		endoliths	0.59±0.00	0.25±0.03	0.29±0.01	0.01±0.01	0.03±0.01	0.04±0.01	7.40±0.49	7.54±0.36	7.33±0.36

Table S2: Sangsawang et al., 2017
