# Supplementary material for: 13C and 15N assimilation and organic matter translocation by the endolithic community in the massive coral Porites lutea
Source: R Soc Open Sci. 2017 Dec 6;4(12):171201. doi: 10.1098/rsos.171201 (PMC5750018; doi:10.1098/rsos.171201)
Supplement: Table S3 [file rsos171201supp3.pdf]

Table S3. ^{13}C atom (%) recorded in healthy and bleached *Porites lutea* (coral tissue and endolithic community) during the two incubations at initial, 12 h (light period), and 24 h (light and dark periods).

incubation	condition	replicates	^{13}C atom (%)		
			initial	12 h	24 h
Addition	healthy	tissue 1	1.0921	1.1319	1.1656
		tissue 2	1.0918	1.1668	1.1262
		tissue 3	1.0916	1.1556	1.1317
		endoliths 1	1.0898	1.1015	1.1082
		endoliths 2	1.0943	1.0966	1.1138
		endoliths 3	1.0928	1.0899	1.0939
	bleached	tissue 1	1.0939	1.0959	1.0951
		tissue 2	1.0921	1.0944	1.0939
		tissue 3	1.0943	1.0971	1.1186
		endoliths 1	1.0871	1.0930	1.0921
		endoliths 2	1.0884	1.0916	1.0896
		endoliths 3	1.0871	1.0894	1.0836
Injection	healthy	tissue 1	1.0945	1.2188	1.2311
		tissue 2	1.0945	1.1841	1.2091
		tissue 3	1.0898	1.1644	1.2454
		endoliths 1	1.0895	1.1821	1.2291
		endoliths 2	1.0867	1.1770	1.2321
		endoliths 3	1.0921	1.1422	1.2696
	bleached	tissue 1	1.0917	1.1231	1.1535
		tissue 2	1.0919	1.1241	1.1812
		tissue 3	1.0881	1.0937	1.1636
		endoliths 1	1.0867	1.2262	1.1959
		endoliths 2	1.0924	1.2138	1.1969
		endoliths 3	1.0884	1.2025	1.1822

Table S3: Sangsawang et al., 2017
